# The Needs of Dentistry

**Published:** 1906-10

**Authors:** L. F. Luckie

**Affiliations:** Birmingham.


### THE NEEDS OF DENTISTRY.

By L. F. Luckie, D. D. S., Birmingham.

Alabama Dental Association.—In the first place, we need more men with the necessary preliminary education to enter the profession—college men, if you please; men who will reason more, think more, and study more before and after they graduate. Can you not see how these men with a broad knowledge would benefit the profession? The educated man, no matter what his eposition in life, is the best laborer.

I stated before this Association at its last meeting that a graduate should study and read to keep up with what is going on in his profession. I repeat it now. We need more intelligent readers. "Reading maketh a full man." Any one who ceases to take an interest in his profession and does not read and increase his knowledge of his profession is a barterer and a trader pure and simple; and this brings me to another need, which is more professionalism and less commericalism. Too many men enter our profession as a business enterprise for the sake of the money that is in it. They give no thought to the professional side of dentistry.

Did you ever hear of a physician who discovered a new treatment of a medicament that was not glad to give it out to his profession, unless he was a quack of some kind? Is this so in dentistry? Let us see. A dentist discovers a treatment for an abscess or some other disease of the oral cavity. Does he publish it for the benefit of his profession? No. He has it patented and prepared in small bottles without even the formlla on the label, and advertises it for sale at an exorbitant price. A phase of commercialism, that is all! My idea of professionalism is to conscientiously treat conditions as we find them, and not take in consideration which would put the most money in our pockets, and charging a fee commensurate with the operation. I believe that dentistry should be a specialty of medicine, and the day is not far distant whes it will be. Why should it not be? The eye, ear, nose, and throat specialist is a graduate of medicine, and there is just as much reason in saying that he must confine his knowledge to those organs as the dentist should confine himself to a mere knowledge of hte teeth. This is a day of reform and advancement, and our profession must advance with other things. Dentistry means more than placing fillings in teeth or restoring lost organs in the oral cavity. It is broader in its scope. The condition of the cavity has a great deal to do with the health of the individual. Because we have put the teeth in good repair does not fully alleviate the condition. We must be able to diagnoses all diseases of the cavity. To instruct our patients as to the best course to pursue for the alleviation of the disease, we should know when to advise them to consult a physician or surgeon; and if the disease comes within the scope of our profession, we should be able to treat the case ourselves, even though it require internal medication. How many of our profession are able to do these things? I believe this broad knowledge is necessary for the intelligent and successful treatment of our patients. If you think that they do not read and converse about the professions, you are mistaken.

I hold dental colleges responsible to a great extent for the position occupied by dentistry at a meeting of the Institute of Dental Pedagagies held at Chicago in 1902 Dr. G. V. Black said: "The future of this Association will be what the dental profession of America makes it, and what they make it will depend largely upon the teaching in the dental schools."

At the same meeting, Dr. Hoff, of Ann Arbor, Mich., said: "The sooner the National Faculties Association adopts as a standard of preliminary education high-school graduation for admission to its schools, the sooner will we began to make such advance in the teaching of theoretical branches as are ideal. I believe that when we demand a high-school diploma for entrance to our schools, we will not be bothered so much about the curriculum, what subjects shall be included, and how much of them shall be taught."

The Association or dental faculty met in 1903 and decided on a four years' course. Some of the profession thought and felt that we were advancing on the right lines. At the following meeting they voted to return to a three1years course. Why was it? Was there not enough in the curriculum to warrant four years? If not, it would not have been much trouble to have found it. Or was it because so many weaklings, fearing a four-years course, dropped out, thereby so decreasing the income of the colleges that a spirit of commercialism prompted the dental faculty to reduce the course to three years? I say, I charge the dental colleges for being responsible to a great extent for the condition in which we find ourselves.

Another need in the profession is fellowship. Although a dentist, I associate more with the members of the medical profession than with my own There is a reason for this. It is not because I think less of my brother dentists, for I would like to affiliate with them; but it is because they do not seem inclined that way, and I do find my friends of the next to the greatest profession in the world to-day congenial and kind. A brother dentist remarked to me only the other day: "There is more ealousy in the dental profession than in any other profession." I do not know but what he was right. Such jealously often takes the form of criticism and abuse. Criticism and abuse do no good, but react on one's self, and often where it is least expected. One should look into and examine one's self first before finding fault with another, for

"There is so much good in the worst of us
And so much bad in the best of us
That it doesn't become any of us
To talk about the rest of us."

Some writer said, and it is a good thing for any one, no matter what his business, to carry with him: "Let thy every word and act be perfect truth uttered in genuine love. Let not the forms of business or the conventional arrangements of society reduce thee into falsehood. Be true to thyself, be true to thy friend, be true to the world."

To recapitulate: First, we need more college men; second, more study and intellectual reading; third, more professionalism and less commercialism; fourth, more fellowship. How can these things be brought about? This way: Make our profession more desirable by a dignified and intelligent bearing. Prove to the outside world that we are thinkers and students, instead of mere mechanics. Cut out the professional jealousy. Do away with petty scraps about little nothings in our Association meetings. Show in our papers research and study, so that we can give to our fellows something on which to think. Have a purpose in view, and let that purpose be to benefit the profession of which we are members. Carlyle says: "Have a purpose in life, and, having it, throw into your work such strength of mind and muscle as God has given you."

